# Verses

**Published:** 1906-10

**Authors:** 


### REACHING HEAVEN.

"Heaven is not reached by a single bound,
But we build the ladder by which we rise
From the lowly earth to the vaulted skies,
And we mount to its summit round by round."

—*J. G. Holland.*

### AUTOMOBILIOUS.

A wealthy old pater-familias
Took on such an air supercilious,
His wife sought a doctor,
Whose answer quite shocked her:
"Needs watching—he's automobilious."

—*Med. Visitor.*

### HAPPINESS.

Talk happiness. The world is sad enough
Without your woes. No path is wholly rough.
Look for the places that are smooth and clear,
And speak of those to rest the weary ear
Of earth, so hurt by one continuous strain
Of human discontent and grief and pain.
—*Ella Wheeler Wilcox.*

### THE FROLIC OF THE MICROBES.

A Phagocyte and Leucocyte
Went to attend the play one night.
The "Micrococci Neoformans"
Preferred a matinee performance;
The farce was Doyen's Cancer Cure,
Which gave delight, you may be sure,
And roars of laughter shook the house
When shown some tumours from a mouse,
The Gonococci, funny chaps!
Gave all the actors hearty claps;
Who never thinking of the cause
Accepted such as pure applause,
The farce stills holds its place on bill,
Actors and play are running still.
—*Medical Times Hospital Gazette.*

### Choice of Transportation.

Ef I wus agwine ter go sum whar
Dere's jess no use een talkin'
I'd tek er donk befo' I'd walk
Kase po ridin' beats good walkin'.

An er waggin er two wheel kyart
Beats hoss-back ridin' on er mule
An ter tek er coach instead of kyar
I'd be er pint blank fool.

Twix rale-rode kyar an oshen bote,
I kno' which I'd wish fer,
Youse dar on lan' ef de biler buss
Ef on de brine, whar is yer?
*B. H. Teague.*

### THE SCRIPTURE VERSUS PASSES.

"Thou shalt not pass."—*Numbers xx.,* 18.
"Suffer not a man to pass "—*Judges iii.,* 28.
"The wicked shall no more pass."—*Nahum i.,* 15.
"Though they roar, yet can they not pass."—*Jer. v.,* 22.
"He paid the fare and went."—*Jonah i.,* 3.

—*Exchange.*

### THE COTTON-BOLL WEEVIL.

De cotin man habs er haard ole time,
His rows am strewed wid evil,
Cool nites, an lice, an rain een line,
An now comes that durn boll-weevil.

Hits er leetle cussed, fly-like bug,
Wid er mout mek like er drill,
An wen de boll gits 'gin dat mug,
Hit sho do damage wid dat bill.

We've tried de birds ter eet de pes',
Put Paris green on boll an plant,
An we ain't a goin' ter let im res',
Fo we've put pun him dat Dago ant,

We're hopin' dat twixt dese two.
Dere'll be er mos' oulastin' scrap,
An een de long run 'twill be true
Hits whipped fum out de cotin crap.

—B. H. Teague.

